# Stevens-Johnson Syndrome complicated by obstructive uropathy, pneumothorax, and pneumomediastinum: a case report and literature review

**DOI:** 10.1186/s41038-019-0153-4

**Published:** 2019-06-11

**Authors:** Damian Bruce-Hickman, Xiao Jiang, Joshua Jin-Ping Thia, Amit Kansal

**Affiliations:** Department of Intensive Care Medicine, Ng Teng Fong Hospital, Singapore, Singapore

**Keywords:** Stevens-Johnson Syndrome, Obstructive uropathy, Ureteric stenting, Pneumothorax, Pneumomediastinum

## Abstract

**Background:**

Stevens-Johnson Syndrome (SJS) is an acute mucocutaneous eruption with blisters of the skin and haemorrhagic erosions of mucous membranes. This report describes air-leak syndrome and obstructive uropathy occurring simultaneously in a teenage patient affected by SJS.

**Case presentation:**

A 17-year-old Malay female with SJS suffered from bilateral pneumothoraces, pneumomediastinum, and obstructive uropathy as early complications of her disease. She required intubation, chest tube insertion, and bilateral ureteric stenting as part of her intensive care management. These extra-cutaneous complications of renal and pulmonary systems were likely secondary to widespread epithelial detachment.

**Conclusion:**

Despite paucity of cases in adult literature, post-renal causes for acute kidney injury must be considered in SJS, especially in the setting of gross haematuria. Bedside point-of-care ultrasonography may be a useful tool for excluding obstructive uropathy. Pneumothorax is a rare but documented complication of SJS in paediatric cases and, to a lesser extent, adult patients. Extra care should be exercised when caring for mechanically ventilated patients suffering from SJS.

## Background

Stevens-Johnson Syndrome (SJS) is an acute mucocutaneous eruption with blisters of the skin and haemorrhagic erosions of mucous membranes. This report describes air-leak syndrome and obstructive uropathy occurring early in the disease course of a teenage patient affected by SJS. The importance is that it is the first report in the medical literature describing these two complications occurring simultaneously in an affected patient. We describe the events during our patient’s stay on the intensive care unit (ICU), provide a literature review of these rare complications, and derive clinically important learning points from our experience.

## Case presentation

A 17-year-old Malay female with no significant past medical history presented with rashes over the neck and trunk and swollen lips. She had been unwell for 2 days prior to hospital attendance with symptoms of fever, sore throat, and running nose and red itchy eyes. She had attended her family physician 1 day before presentation to hospital and had been prescribed paracetamol, dequalinium lozenges, loratidine/pseudoephrine nasal spray, and co-amoxiclav tablets, of which she had taken two doses.

**Fig. 1:**
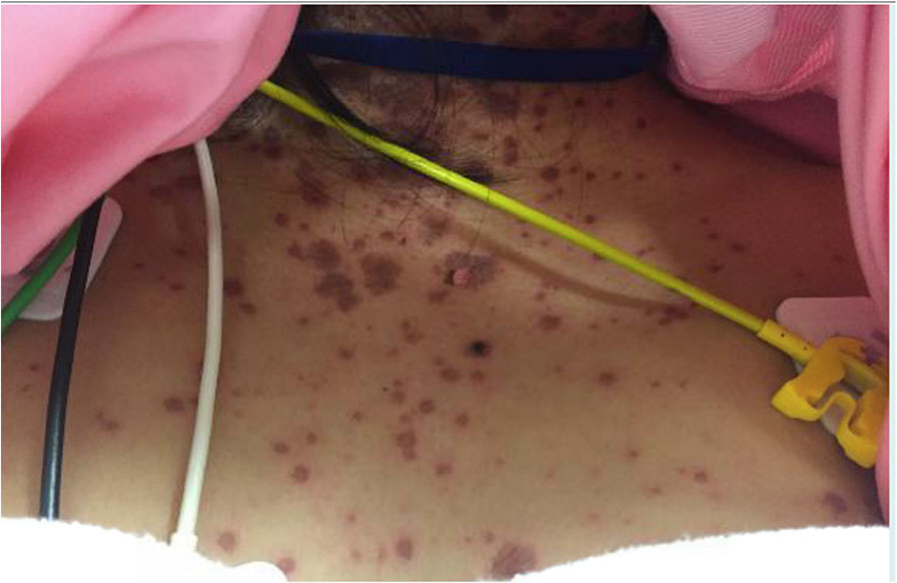
Blisters on patient’s upper chest demonstrating positive Nikolsky’s sign

**Fig. 2:**
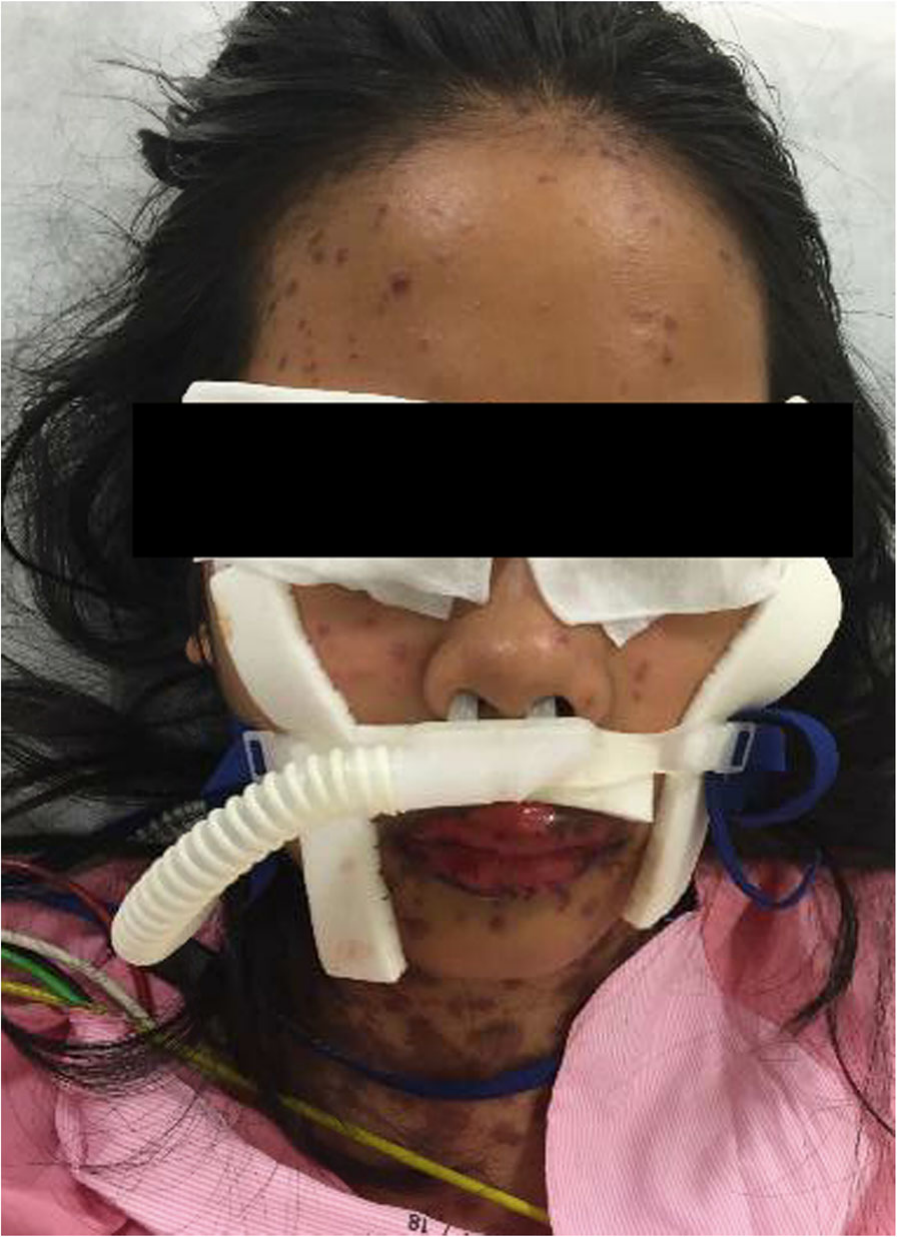
Lesions distributed over the face and mucosal erosions of the lips

**Fig. 3:**
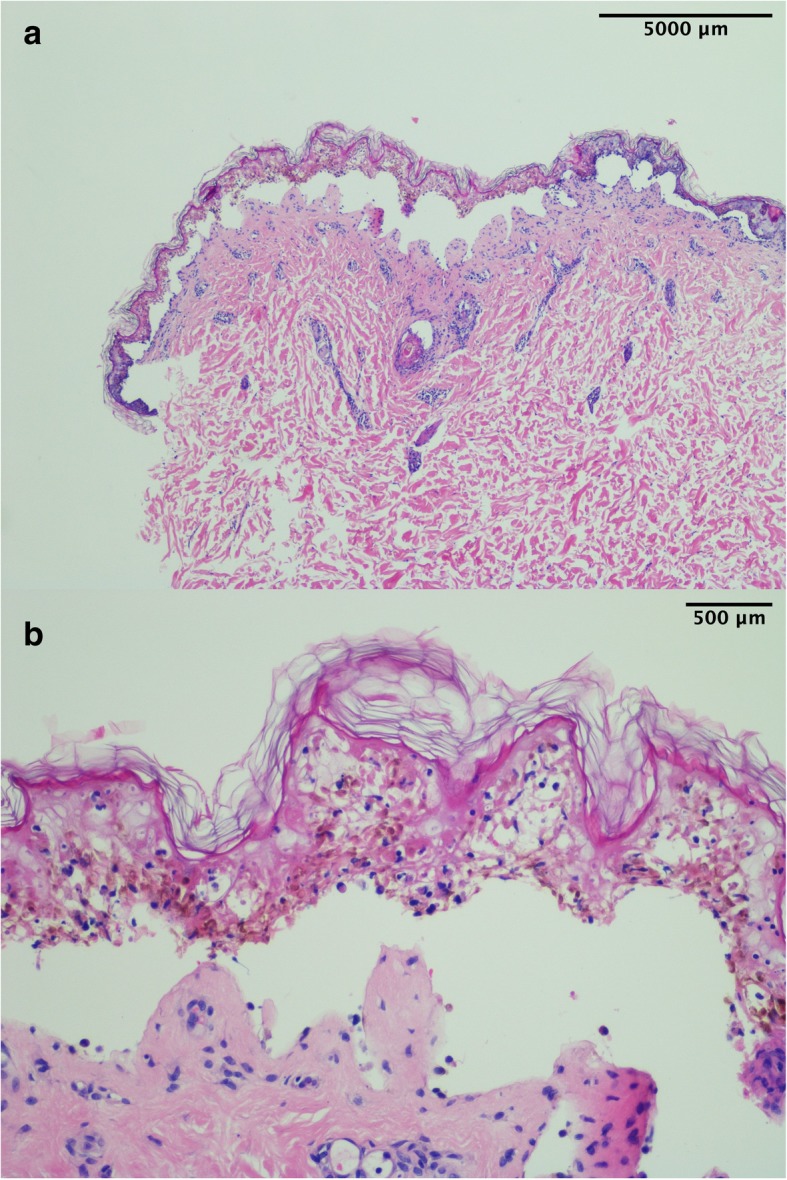
Histological findings from skin biopsy. **a** Low-power image showing sub-epidermal splitting. **b** Higher-power image shows splitting with necrotic keratinocytes in the epidermis, accompanied by apoptotic debris

Inspection of the patient’s skin revealed scattered dusky, flaccid blisters demonstrating positive Nikolsky’s sign (Fig. 1). These were distributed over the face (including hairline and scalp), neck, anterior trunk, and back (Fig. 2). Most of her arms and all of her legs were spared. There was no evidence of secondary cutaneous infection. Mucosa erosions were seen on the lips as well as the labial surfaces. Opthalmic examination demonstrated injected conjunctiva with pseudomembranes of the upper and lower palpebral conjunctiva. Corneal examination showed a 6-mm epithelial defect on the right side. Skin punch biopsy demonstrated sub-epidermal splitting, necrotic keratinocytes in the epidermis, and apoptotic debris (Fig. 3a, b). These clinical and histological findings were consistent with a diagnosis of Stevens-Johnson Syndrome. Severity of illness score for toxic epidermal necrolysis (SCORTEN score) on admission was 2. The trigger for her SJS remains uncertain. ALDEN scoring for all drugs was − 3; onset of red eye symptom was taken as probable index day [1]. Infectious screening for human immunodeficiency virus, respiratory viral panel (influenza A and B, respiratory syncytial viruses A and B, coronaviruses), Mycoplasma (Acute titre), and Epstein-Barr virus serology were negative. Serum herpes simplex virus (HSV) IgM was positive, but nasopharyngeal HSV 1 and 2 cultures were negative.

**Fig. 4:**
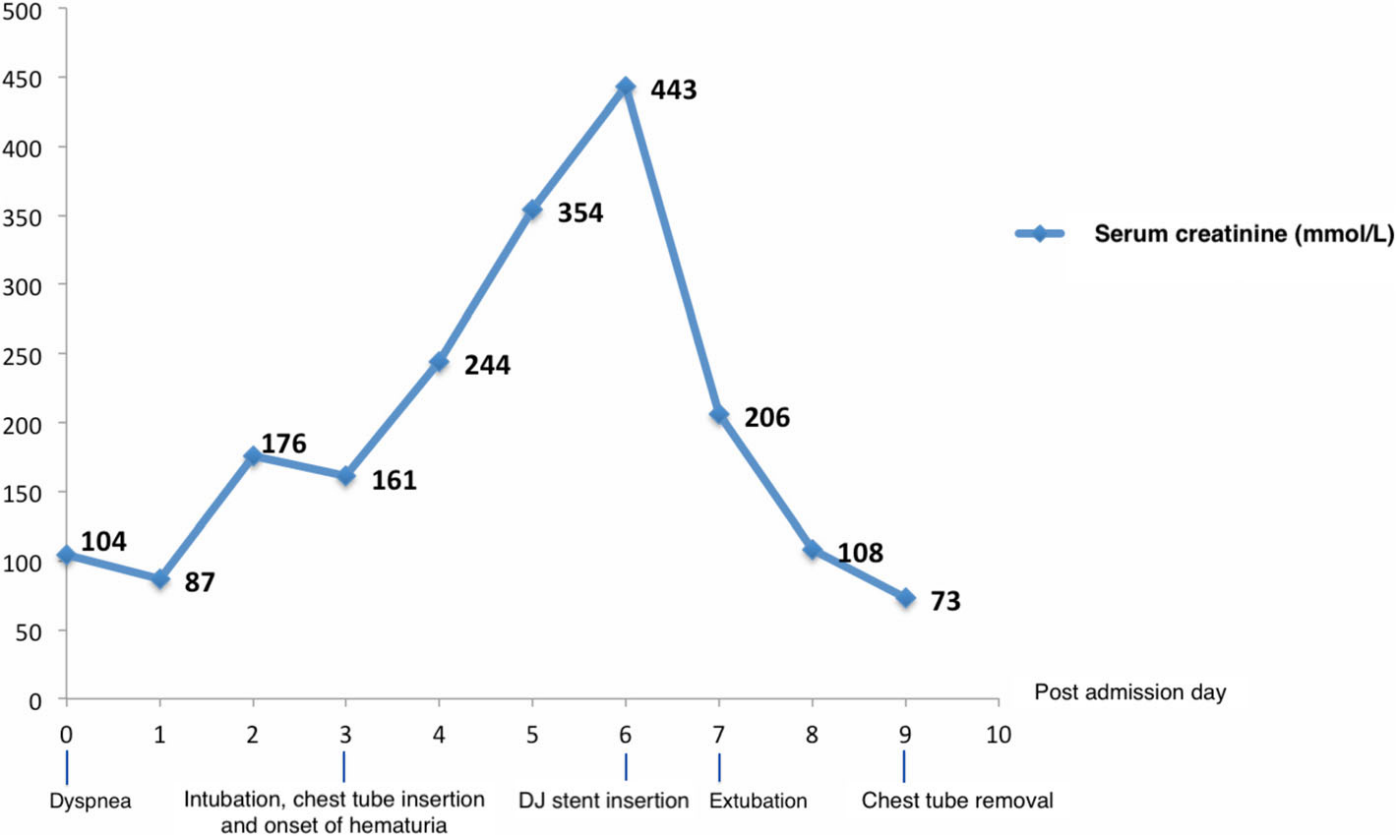
Timeline of significant events during patient’s hospital course

The patient was admitted to our ICU with tachycardia and tachypnoea, although blood pressure and peripheral oxygen saturation were within normal limits at the time of admission. The patient subsequently developed significant pulmonary and renal complications which are described below. A timeline of significant events and trend of renal function is outlined in Fig. 4.

### Urology complications

**Fig. 5:**
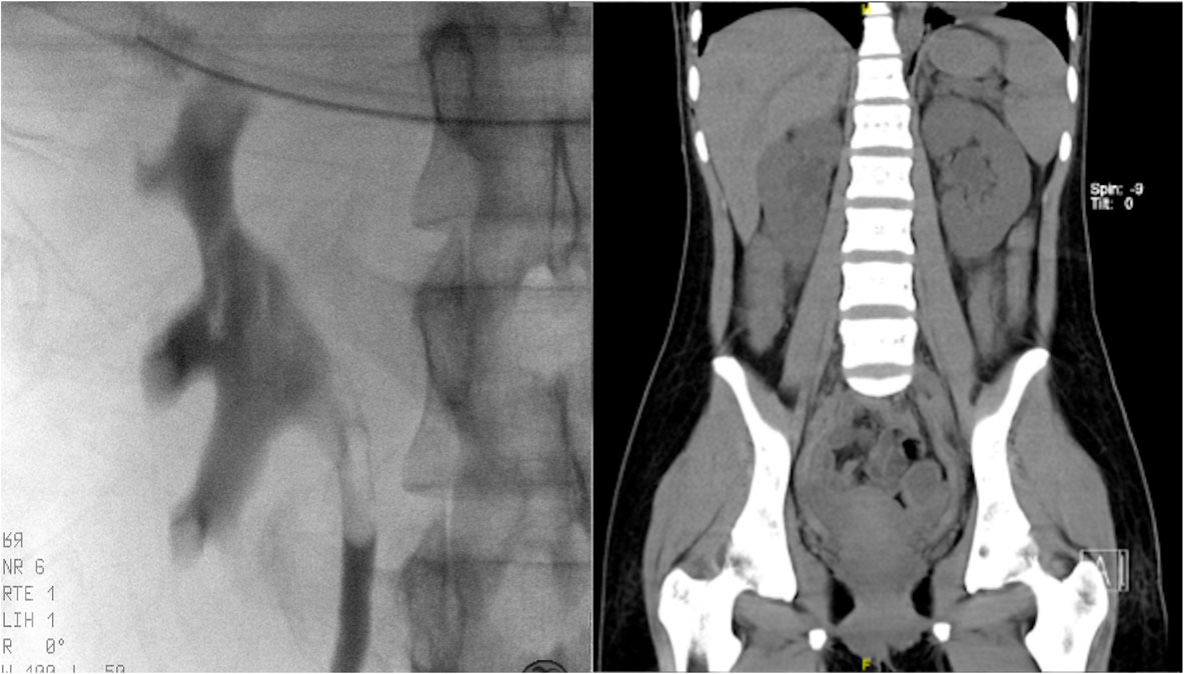
CT imaging showing bilateral hydronephrosis and mild hydro-ureters (right); fluoroscopy imaging confirming filling defect (left)

Blood tests on admission showed acute kidney injury (creatinine 104 mmol/L). Our patient’s serum creatinine continued to worsen during admission despite adequate fluid replacement. Gross haematuria was noted from urinary catheter on the third day of admission. Urine analysis was negative for casts with 90% isomorphic red blood cells, suggesting urological origin of haematuria. On the fifth day of admission, her urine output dropped significantly. Bedside ultrasound revealed bilaterally enlarged collecting system, and subsequent CT-KUB confirmed bilateral hydronephrosis and mild hydro-ureters with the presence of dependent debris in pelvicalyceal system and ureters (Fig. 5, right). Rigid cystoscopy showed normal urethra, but bleeding from both ureteric openings and clots in the collecting system. Fluoroscopy confirmed filling defects (Fig. 5, left). Bilateral ureteric stents were deployed intra-operatively. Subsequently, her urine output improved dramatically with immediate improvement in creatinine and resolution to baseline within a few days of stenting.

### Pulmonary complication

**Fig. 6:**
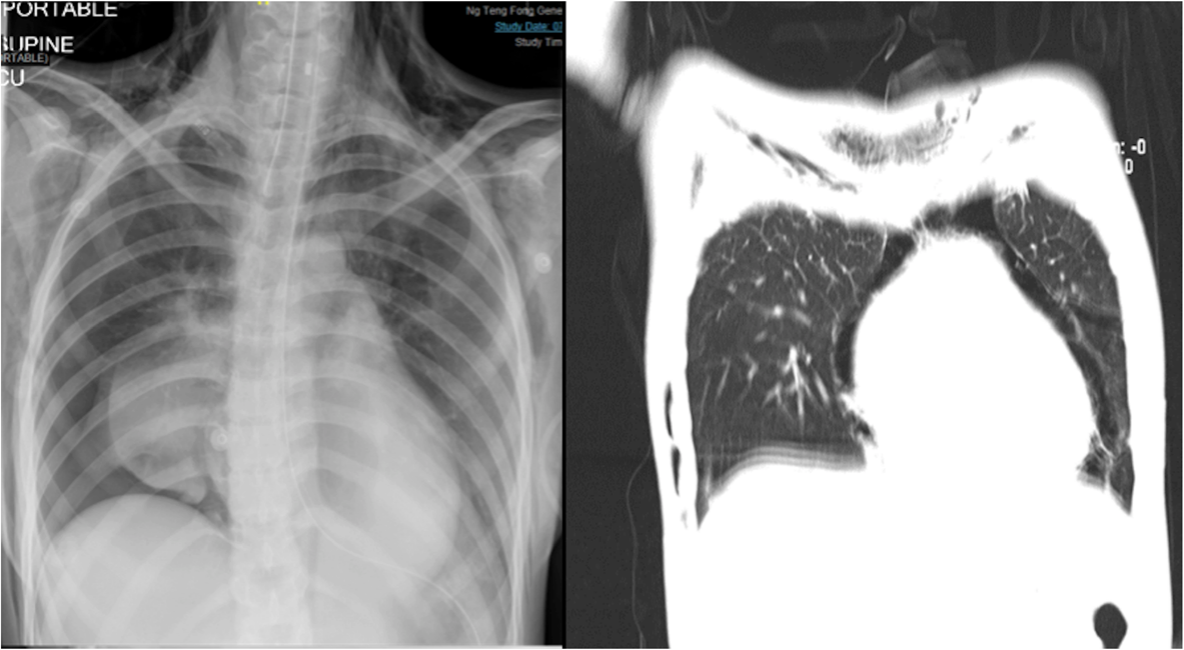
Chest radiograph showing subcutaneous emphysema, pneumothoraces, and pneumomediastinum (left); CT thorax demonstrating pneumomediastinum (right)

Our patient had respiratory distress on admission, and nasoendoscopy revealed mild arytenoid and supraglottic swelling. Respiratory condition worsened on day 2 of admission; the patient became progressively more tachypnoeic with increasing secretion load and required supplemental oxygen. Due to work of breathing, she was initially trialled on non-invasive ventilation (NIV) which was soon switched to high-flow nasal cannula (Fi02 0.3, flow rate 30 L/min) in view of inability to clear secretions whilst on NIV. Twenty-four hours later, at the end of day 3 of admission, she developed sudden onset severe respiratory distress with desaturation and required intubation and mechanical ventilation. Clinical examination demonstrated extensive subcutaneous emphysema. Chest X-ray confirmed presence of right-sided pneumothorax, small left-sided pneumothorax, and pneumomediastinum (Fig. 6, left). A chest tube was inserted for the right-sided pneumothorax. Pneumomediastinum and contralateral pneumothorax remained stable on several follow-up images throughout the hospital stay, including CT scan for characterisation (Fig. 6, right). Upper gastrointestinal endoscopy excluded mucosal tear in the pharynx or oesophagus. She was successfully extubated on day 7, and chest tube was removed on day 9.

## Discussion

SJS is an acute mucocutaneous eruption with blisters of the skin and haemorrhagic erosions of mucous membranes. The cause of SJS in this patient’s case remains unclear. Although case reports suggest that a single dose co-amoxiclav is sufficient for eruption [2], application of a stringent definition of “probable index day” shows that her SJS process had likely already started prior to medication exposure [3]. Rather, prescription of co-amoxiclav and symptomatic medications were reflective of prodromal symptoms of SJS that were already marking disease onset (protopathic bias) [4]. SJS is driven by cytotoxic T cell destruction of the epidermis. T cells produce cytolytic proteins such as granulysin. The link between drug/infectious agent exposure and T cell-mediated destruction of the epidermis remains poorly delineated. Therapeutic principles for management of SJS include removal of culprit agent, multi-disciplinary care of the patient, and transfer of patient to tertiary care in severe cases. Meticulous daily attention to involved mucosal surfaces, pain requirements, fluid demands, thromboprophylaxis, and calorific requirements are the tenets of ICU care. Active therapy may be instigated as part of expert multi-disciplinary decision making. We point readers to current guidelines for further reading [5].

This case report describes a combination of acute renal failure and acute respiratory failure in a patient with otherwise relatively limited skin manifestations of SJS. Extensive literature search done independently by all the listed authors revealed only one other case with a similar presentation albeit detected only at autopsy; a Spanish case report from paediatric population showed renal and pulmonary epithelial sloughing on post-mortem examination [6]. The unfortunate 9-year-old female patient died after developing massive endobronchial haemorrhage and haematuria with anuria. Autopsy demonstrated normal glomeruli tubules and interstitium, but foci of epithelial necrosis in the urinary tract, haemorrhagic ureteric submucosa, and total necrolysis of the epithelium of all small- and medium-sized bronchial tubules are consistent with the observed clinical course in our report.

### Post-renal acute kidney injury in SJS

Incidence of renal failure is high in SJS and is an independent predictor of mortality [7]. In a retrospective analysis of 96 patients with SJS in Taiwan, Hung et al. demonstrated high frequency of acute renal failure (20.8%), the need for temporary dialysis (3.1%), and long-term dialysis (1%) [8]. The authors attributed causation of acute renal failure equally between prerenal azotemia and intrinsic kidney insult without any mention of post-renal aetiology. Similarly, there has been no mention of obstructive uropathy as a cause of acute renal failure in patients with SJS, despite a significant number of published case series [8–11].

Although microscopic haematuria is reported in up to 23% of cases [12], frank haematuria is uncommon. A urological series in paediatric population described frank haematuria in one patient only, attributed to a genital lesion [13]. A separate 20-year retrospective study at a Thai paediatric tertiary referral centre recorded frank haematuria with concurrent rising creatinine again in only one patient [14], although there was no mention of etiopathogenesis.

Epithelial detachment severe enough to result in eventual renal dysfunction from an obstructive cause is rare and has been described in only a very few case reports [6, 15, 16]. Baccaro et al. reported the first published case of an adult toxic epidermal necrolysis patient with ureteropelvic mucosa damage, haematuria, extensive ureteral mucosal sloughing, and acute renal failure secondary to ureteral obstruction and perforation [15]. A subsequent case report described a case of SJS with ureteropelvic mucosal involvement resulting in gross haematuria [16]. Further examination of the urinary sediment with the phase-contrast microscope indicated a urological cause for the observed haematuria, and a plain and enhanced CT of the whole abdomen confirmed thickening and enhancement of the walls of the renal pelvis and ureter, although ureteroscopy was not performed. The aforementioned post-mortem study in a 9-year-old girl who died of respiratory and renal complications demonstrated haemorrhagic ureteric submucosa with normal glomeruli, tubules, and interstitium [6].

### Air-leak syndrome

Although respiratory failure has been documented in cohorts of patients with SJS [17, 18], air-leak syndrome (pneumomediastinum and/or pneumothorax) is a rare complication. Notably, in a 4-year-long retrospective series of 221 adult cases of SJS where chest radiographs were monitored as part of the study protocol, there was not a single record of pneumothorax or pneumomediastinum [18]. A Japanese 6-year registry of adult and paediatric cases of SJS described mediastinal and subcutaneous emphysema as observed complications, but did not comment upon the incidence of air-leak nor age of patients involved [19]. Literature search yielded only seven reported cases of air-leak; the majority are paediatric cases [20–25], with only one case described in a young adult [26]. Air leak presented as pneumomediastinum in all of these cases, with subcutaneous emphysema documented in six of the seven cases. Pneumothorax was described in three cases only: unilateral in two cases and bilateral in one case.

The aetiology for air leak/pneumothorax in the setting of SJS is unclear. Patients in the previous case series [17, 18] demonstrated severe epithelial injury and bronchial mucosal detachment on bronchoscopic evaluation. Pathologically, injury was characterised by denuded basement membrane. The ulceration remained limited to epithelial surface, whilst the basement membrane and lamina propria remained intact.

A likely hypothesis for air leak is that mucosal debris causes obstructive airway pathology leading to air trapping and rupture of alveoli. Pneumomediastinum is then the consequence of released alveolar air dissecting through the pulmonary interstitium along the broncho-vascular sheaths toward the pulmonary hila, into the mediastinum [27]. This hypothesis may also explain the predominance of paediatric cases due to smaller calibre airways. Forceful coughing due to hypersecretion and irritation from bronchial denudement may also contribute to alveolar rupture. Our patient had thick bronchorrhea and difficulty managing her secretions prior to respiratory deterioration. Bronchorrhea in the setting of SJS is described as clear yellow fluid, without the presence of any bronchial casts and may represent part of the exudative process of SJS rather than secondary to denudement [17]. Mechanical ventilation could be a contributing factor in our patient, although previously published case reports had air leak at presentation to hospital and therefore unrelated to the onset of mechanical ventilation. In the only other published adult case report of pneumomediastinum/pneumothorax in SJS, air leak occurred prior to the onset of mechanical ventilation [26].

## Conclusions

Renal and respiratory involvement in SJS has been documented in SJS cohort studies; however, air leak and obstructive uropathy are rare complications due to systemic epithelial sloughing. Acute care physicians should be aware that significant organ involvement can occur despite relatively limited skin manifestations. Post-renal causes for acute kidney injury must be considered in Stevens-Johnson Syndrome, especially in the setting of gross haematuria and bedside point-of-care ultrasonography may be a useful tool for excluding obstructive uropathy. Pneumothorax is a rare but documented complication of SJS in paediatric cases and, to a lesser extent, adult patients. Extra care should be exercised when caring for mechanically ventilated patients.

## Abbreviations

CT-KUB: Computed tomography of kidneys, ureters, and bladder
HSV: Herpes simplex virus
NIV: Non-invasive ventilation
SJS: Stevens-Johnson Syndrome
